# Supplementary figures and images for: The head turn paradigm to assess auditory laterality in cats: influence of ear position and repeated sound presentation
Source: PeerJ. 2017 Oct 24;5:e3925. doi: 10.7717/peerj.3925 (PMC5659213; doi:10.7717/peerj.3925)

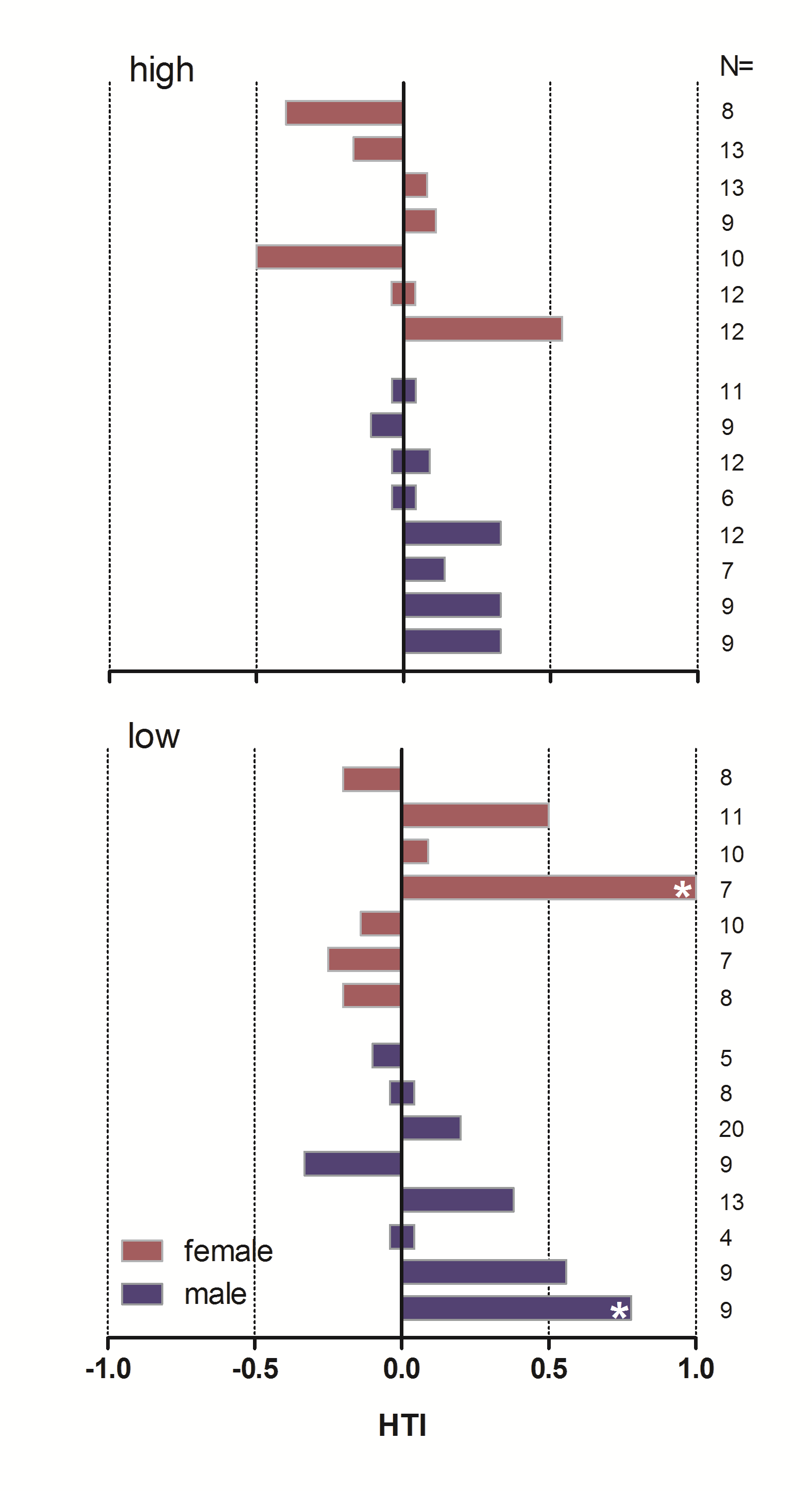

Supplement: Figure S1 — Given are the individual values for males (blue) and females (red) in response to 25 playback presentations. The number of head turns is indicated to the right. Exact binomial test ∗p < 0.05. [file peerj-05-3925-s001.png]
